# Supplementary material for: Transcripts and genomic intervals associated with variation in metabolite abundance in maize leaves under field conditions
Source: BMC Genomics. 2025 May 1;26:434. doi: 10.1186/s12864-025-11580-3 (PMC12046723; doi:10.1186/s12864-025-11580-3)

Supplementary Figure S1

Title:

Supplementary Figure S1. Correlation between 26 metabolite abundance in two replicates of 47 maize genotypes.

Legend:

26 metabolites quantified in a maize diversity panel. REP1 and REP2 correspond to two replications. Metabolites were quantified in two replicates of 47 genotypes.

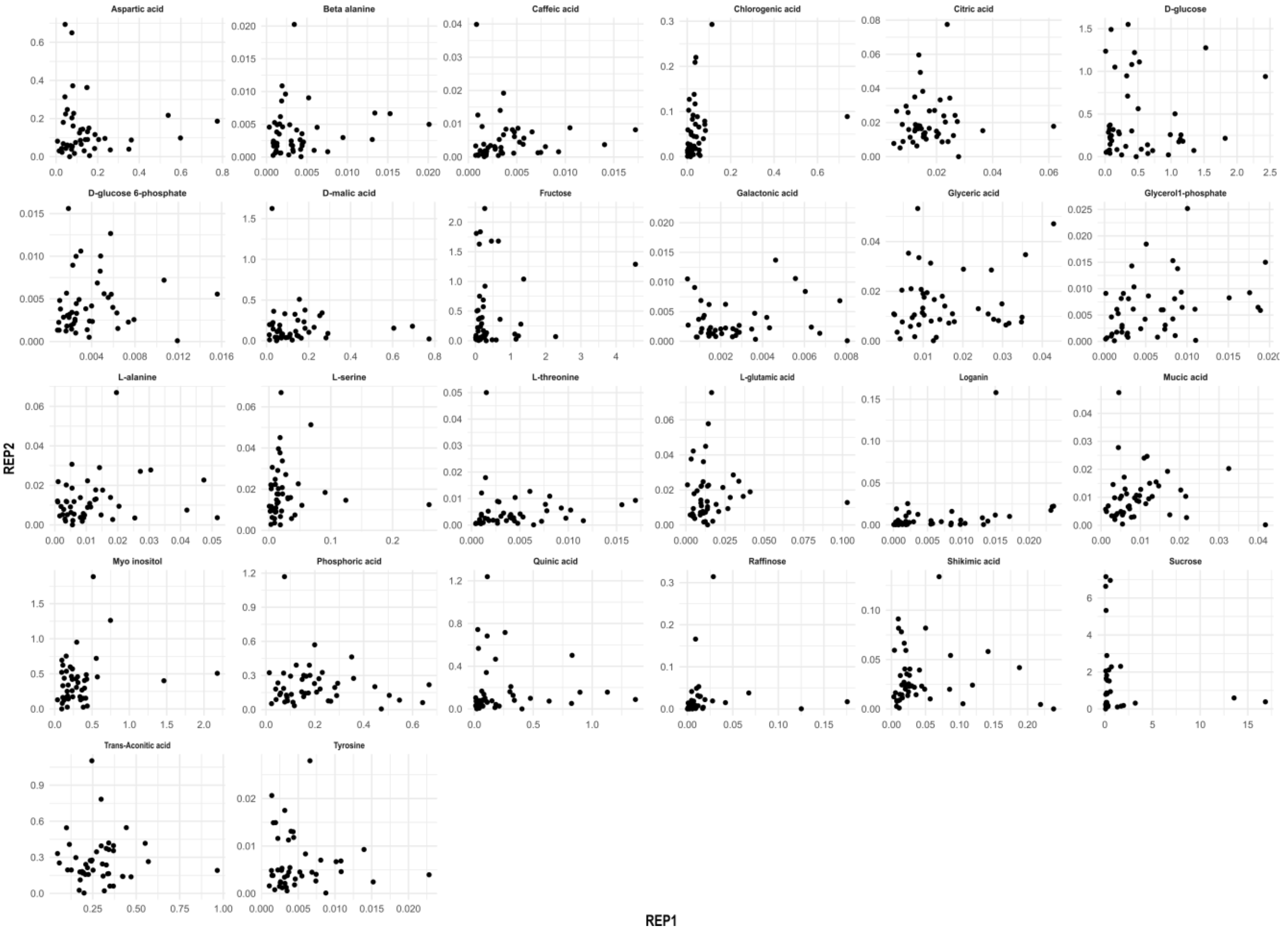

Supplementary Figure S2

Title:
Supplementary Figure S2. Estimated repeatability of 28 whole plant phenotypes used in this study.

Legend:
Repeatability is defined as the proportion of total variance in metabolite abundance which can be explained by genotype in a dataset of 47 maize genotypes sampled and analyzed twice independently from different plants in the same field.

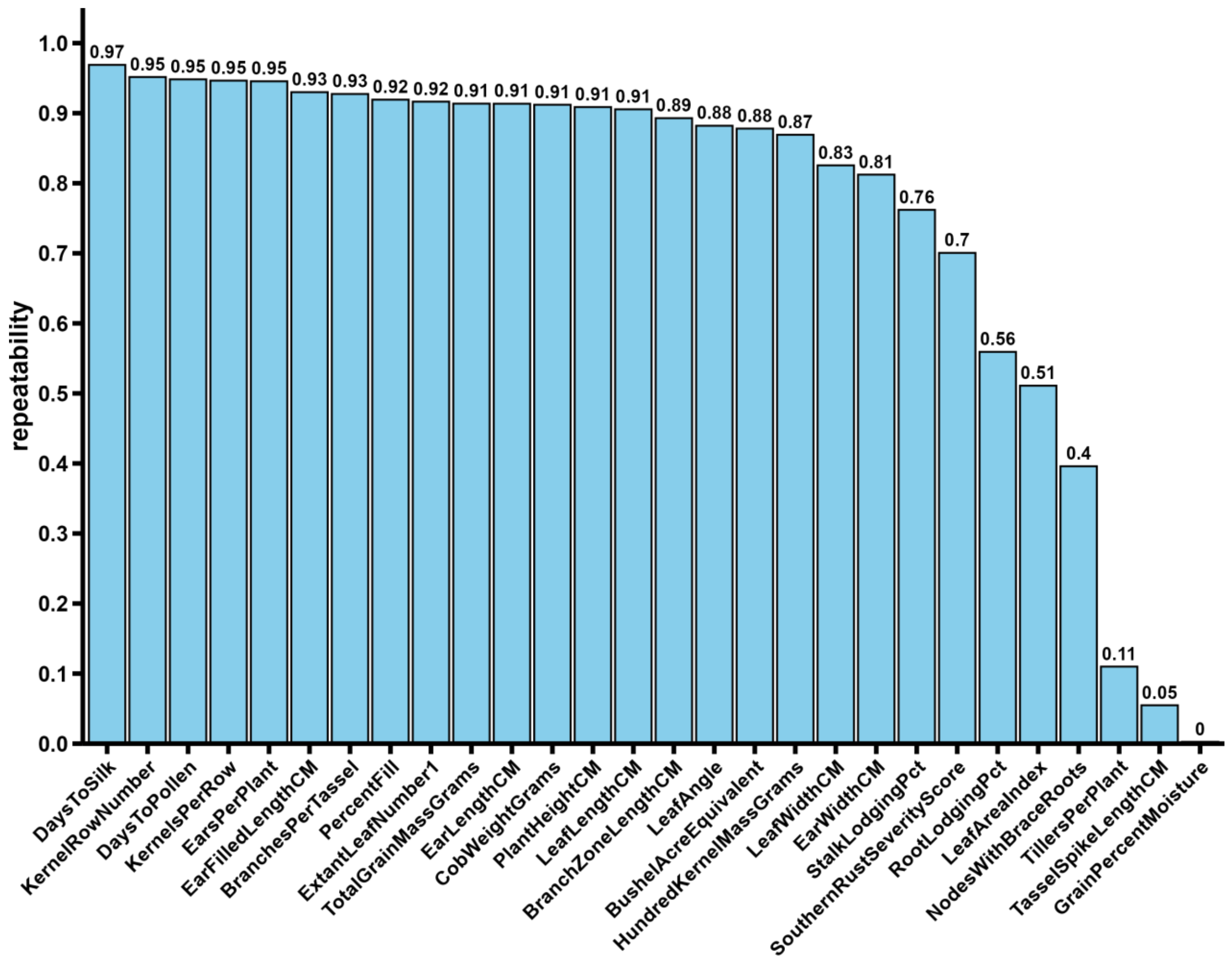

Supplementary Figure S3

Title:
Supplementary Figure S3. Estimated repeatability of ten hyperspectral leaf reflectance derived latent variables used in this study.

Legend:
Repeatability is defined as the proportion of total variance in metabolite abundance which can be explained by genotype in a dataset of 47 maize genotypes sampled and analyzed twice independently from different plants in the same field.

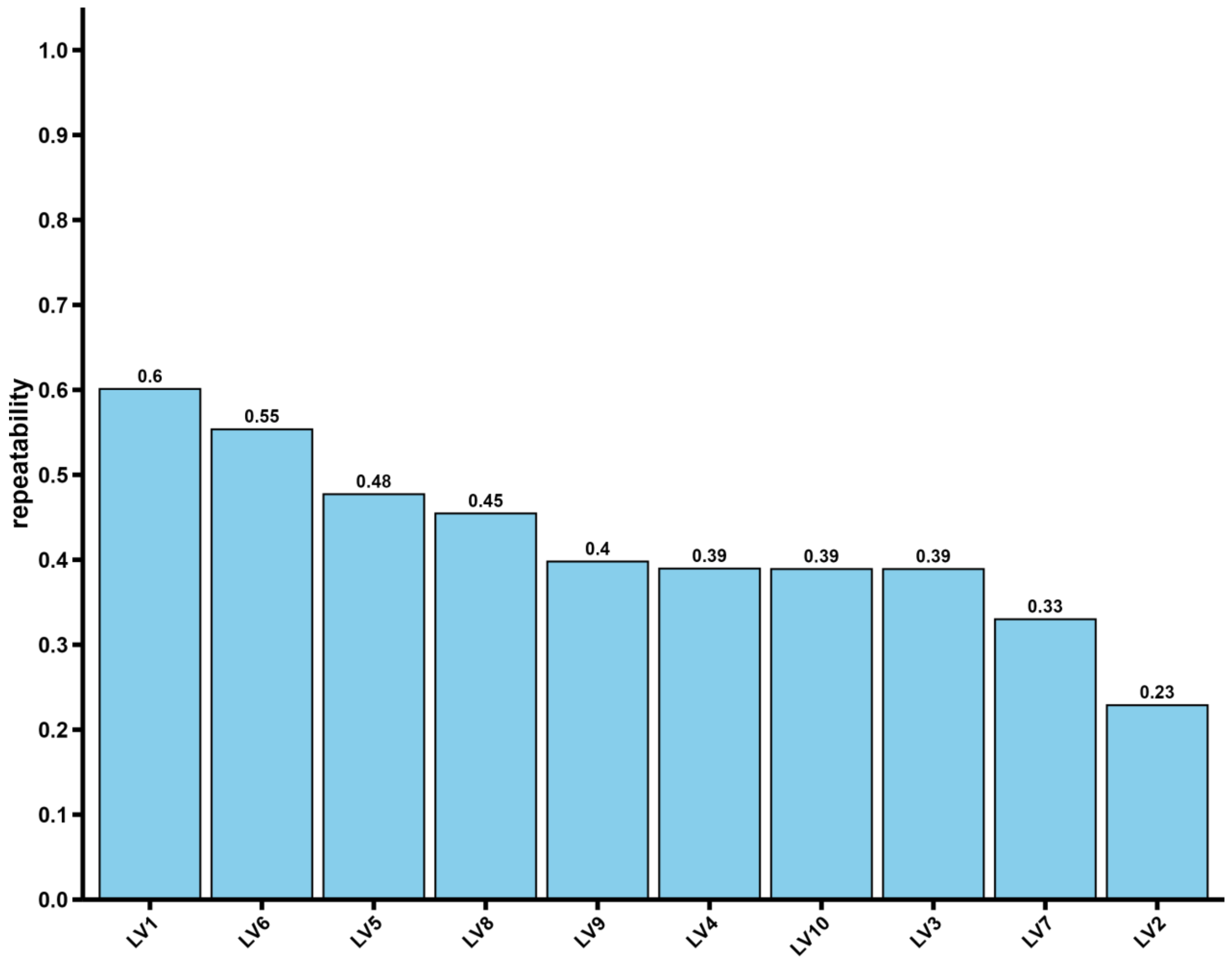

Supplementary Figure S4

Title:
Supplementary Figure S4. Estimated repeatability of three photosynthesis-related traits used in this study.

Legend:
Repeatability is defined as the proportion of total variance in metabolite abundance which can be explained by genotype in a dataset of 47 maize genotypes sampled and analyzed twice independently from different plants in the same field.

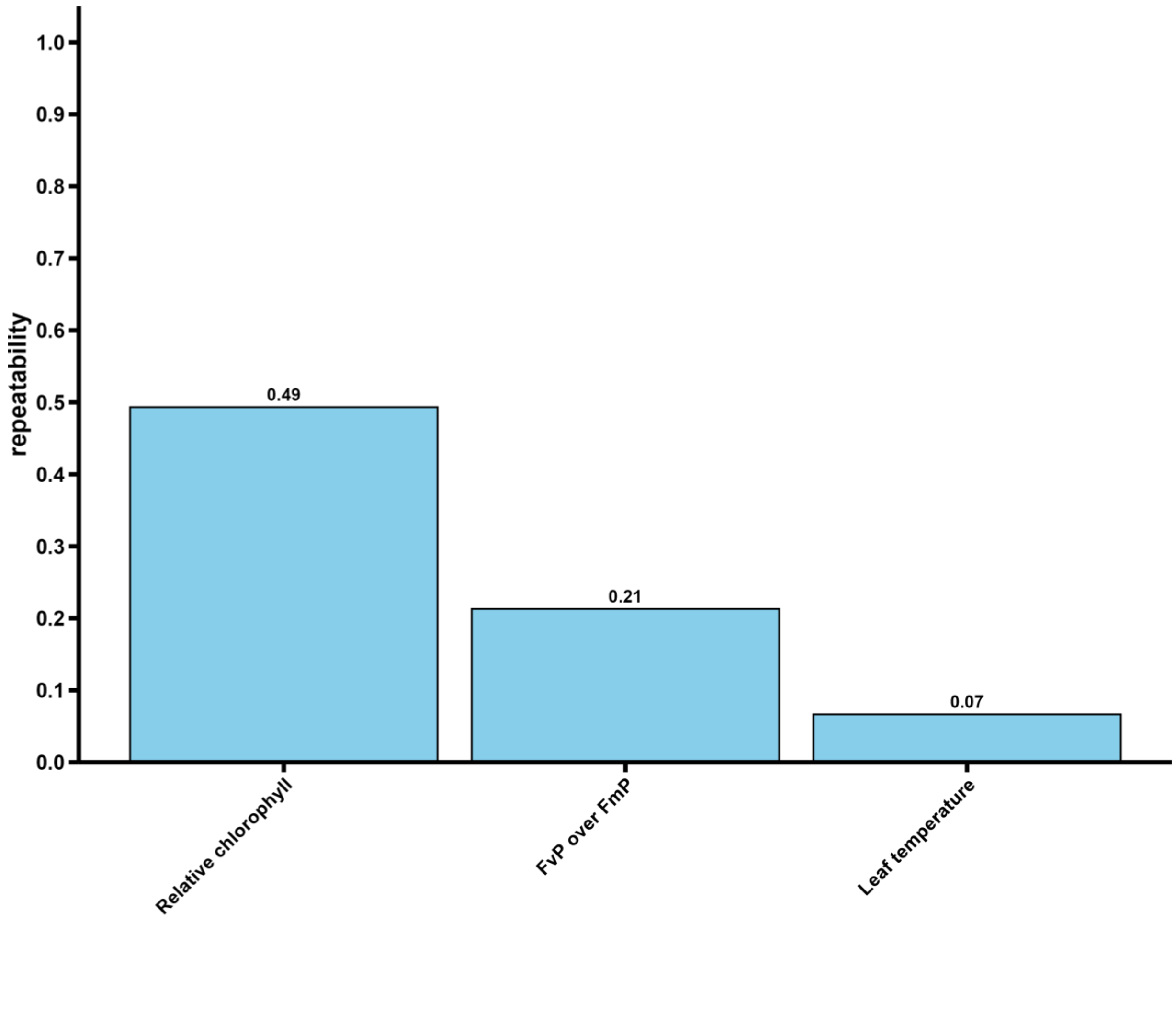

Supplementary Figure S5

Title:
Supplementary Figure S5. Distribution of scores for the first two principal components of variation in metabolite abundance in a maize diversity panel (n = 795 samples).

Legend:
Different colored marker shapes represent subpopulation identity, with abbreviations as follows: SS = Stiff Stalk, IDT = Iodent, NSS = Non-Stiff Stalk. Subpopulation classifications are based on Torres et al. (2024).

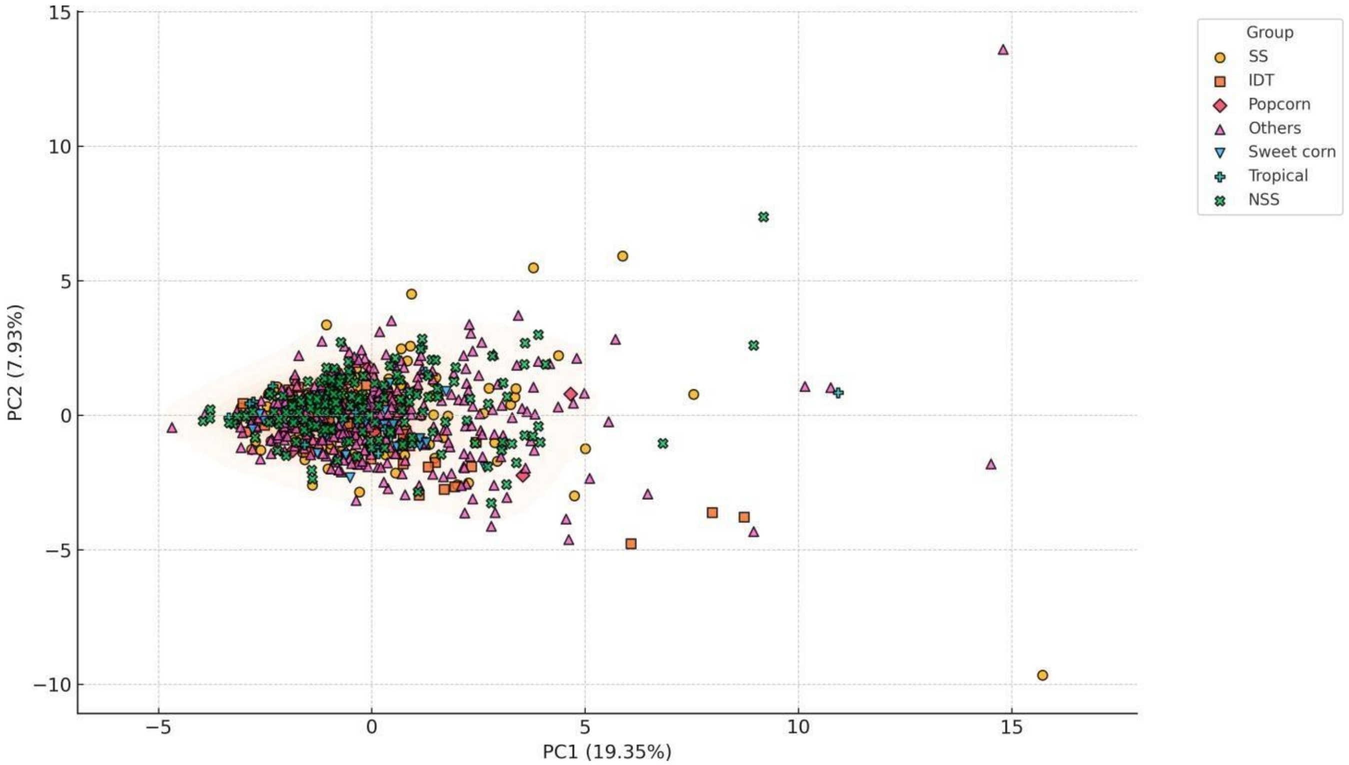

Supplementary Figure S6

Title:
Supplementary Figure S6. Correlation between the variation of 26 metabolite abundance quantified in a maize diversity panel (n = 795 samples).

Legend:
Dark red squares indicate a strong positive correlation, while dark blue squares indicate a strong negative correlation and lighter colors suggest weaker correlations.

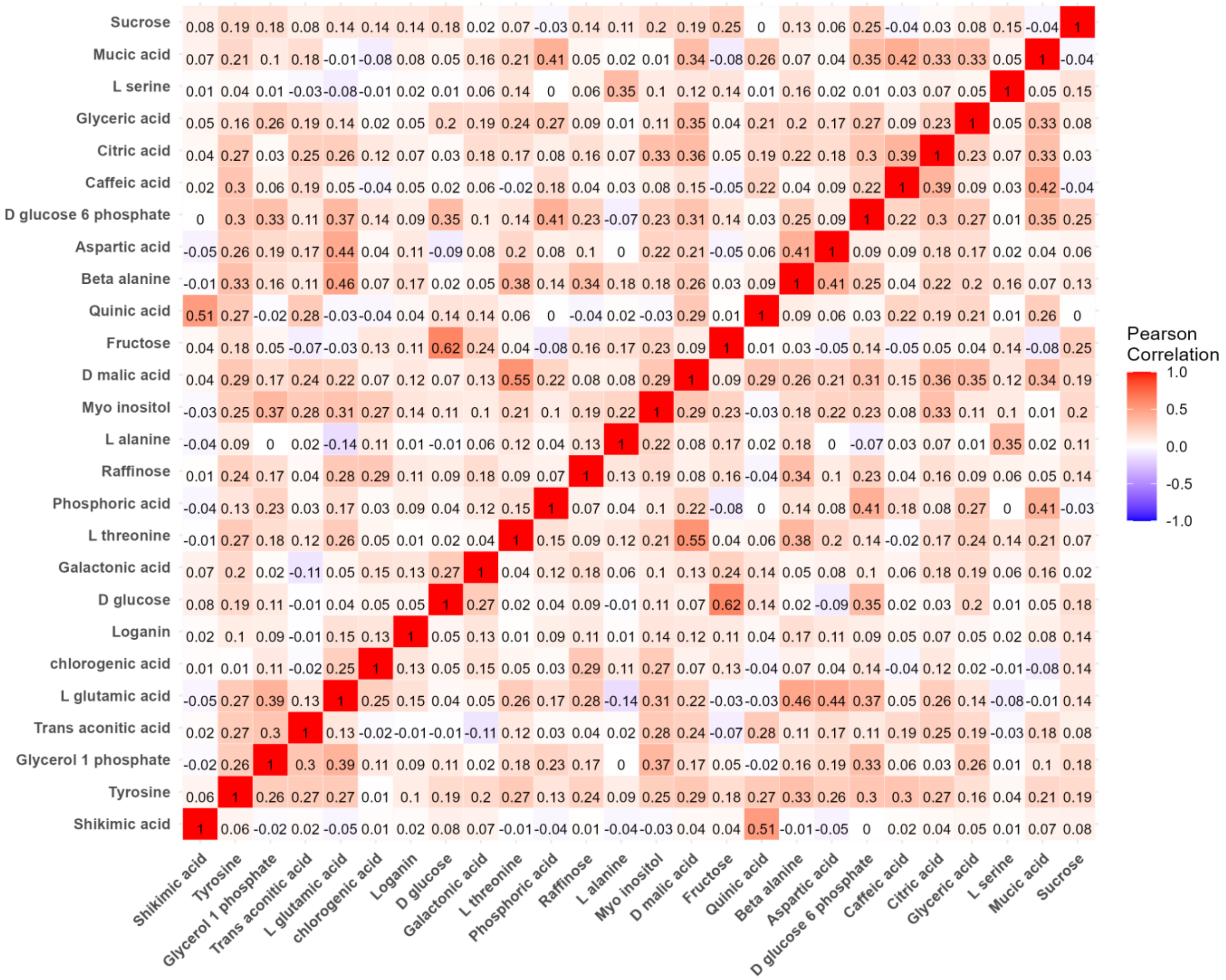

Supplementary Figure S7

Title:
Supplementary Figure S7. Correlation between the variation of 26 metabolite abundance and 41 non-metabolite traits quantified in a maize diversity panel (n = 795 samples).

Legend:
Dark red indicates a strong positive correlation, while dark blue indicates a strong negative correlation, with lighter colors suggesting weaker correlations. The size and fragment of the circles inside each cell indicate the strength and direction of the correlation where larger filled circles represent stronger correlations, while smaller or partially filled circles represent weaker correlations.

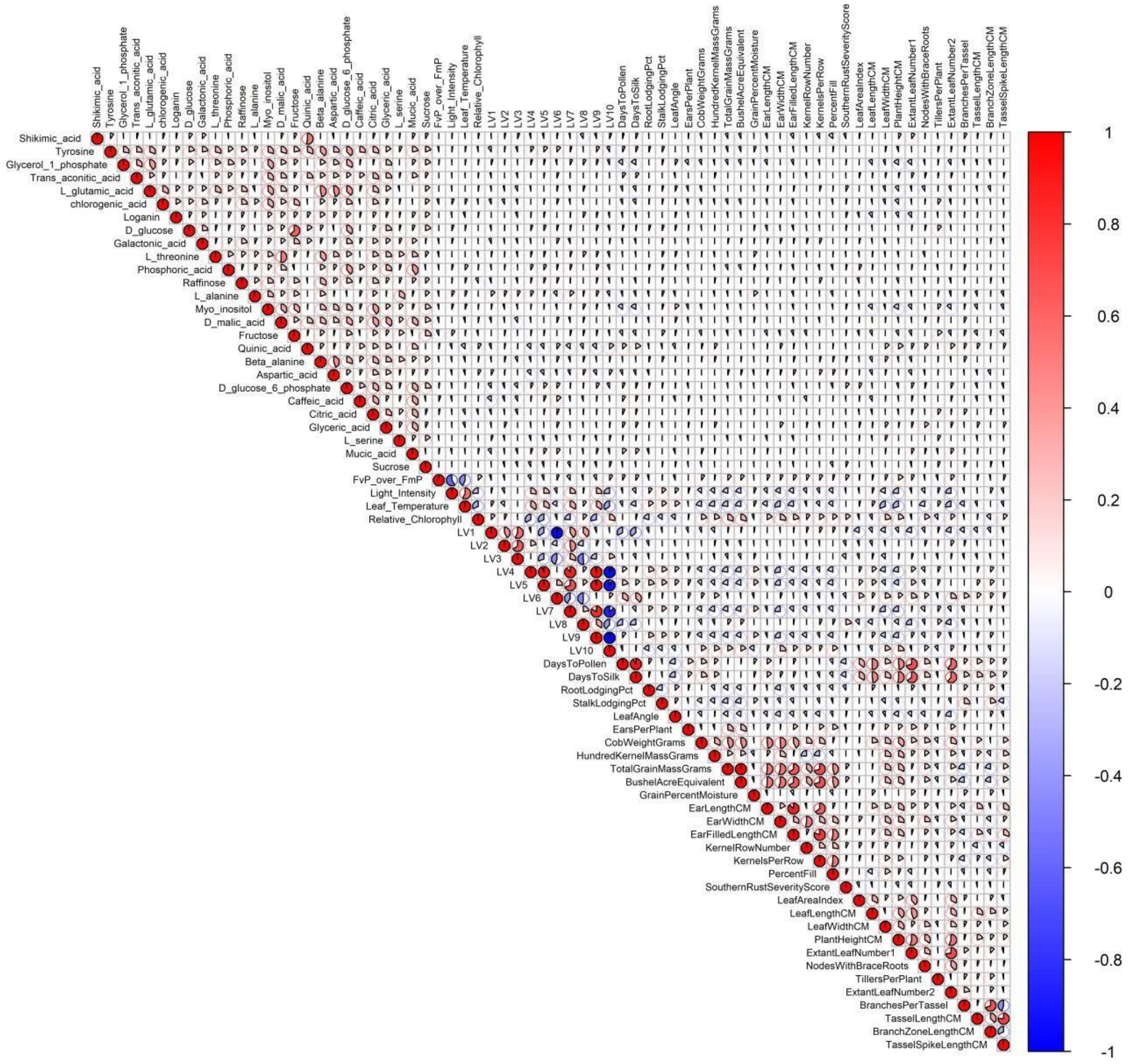

Supplementary Figure S8

Title:
Supplementary Figure S8. Linkage Disequilibrium (LD) heatmap for seven significant trait-associated SNPs highlighted with closest gene models in Figure 2.

Legend:
The green cross marks the genomic position of the candidate gene model and the blue cross marks the genomic position of trait-associated SNP shown in Figure 2.

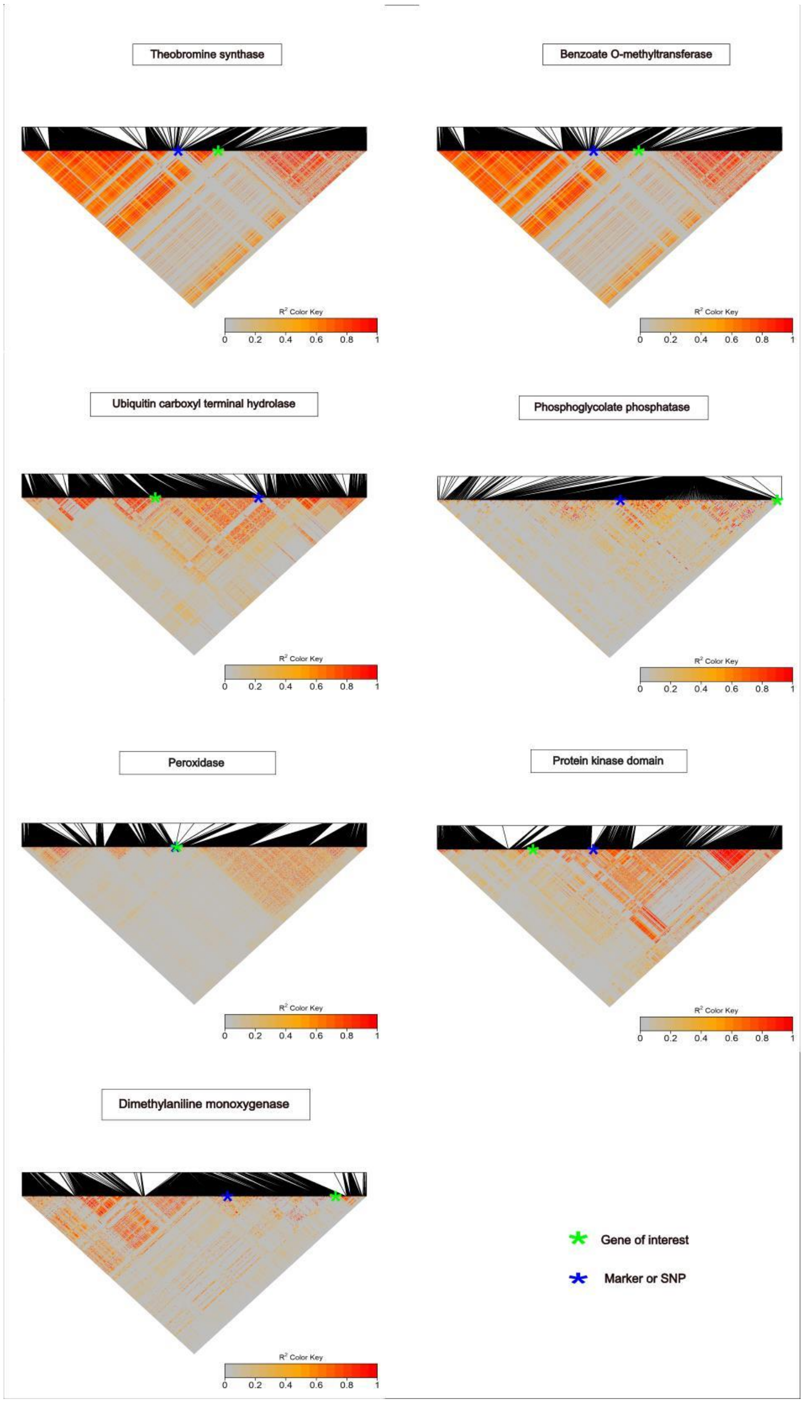

Supplementary Figure S9

Title:
Supplementary Figure S9. Correlation between the abundance of three metabolites and gene expression for significant genes identified via transcriptome-wide association studies, as shown in Figure 3.

Legend:
This figure shows the correlation between the abundance of three selected metabolites and the expression levels of genes identified as significantly associated through transcriptome-wide association studies (TWAS). Correlation coefficients and significance levels are represented for each gene-metabolite pair, illustrating the strength and direction of gene-metabolite associations.

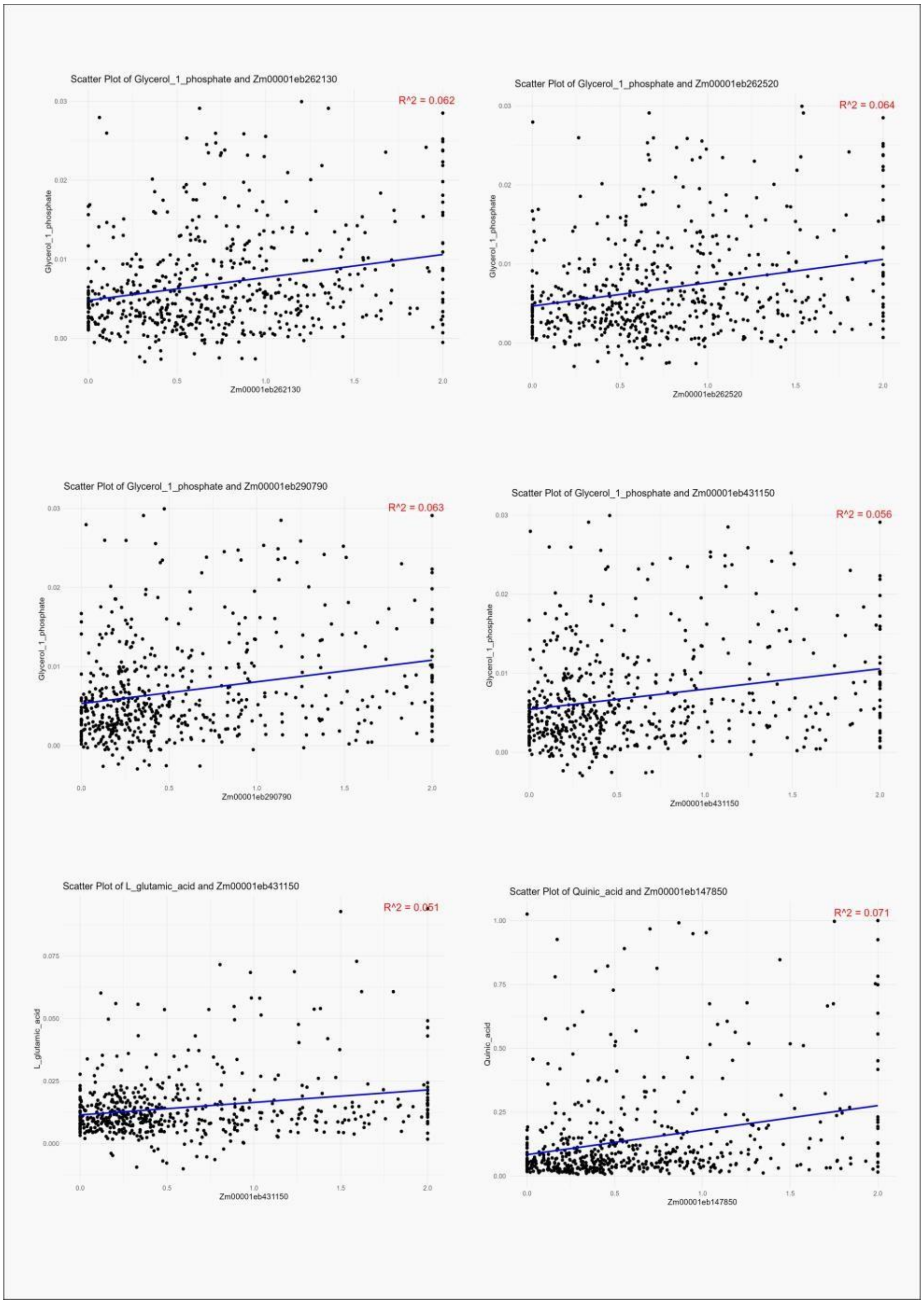

Supplementary Figure S10

Title:

Supplementary Figure S10. Genes identified with higher feature importance in the random forest (RF) regressor model to predict the abundance of three metabolites in a maize diversity panel.

Legend:

The RF model was trained to predict the abundance of three metabolites out of 26 metabolites in the study based on the significant gene expression-trait associations identified in Figure 3. The number of gene models was selected with higher feature importance than a threshold that correspond to a false discovery rate of approximately 0.05 based on a comparison of the features important scores reported for shuffled datasets for each metabolite. The numbers inside the displayed bar charts represent the feature importance assigned to the gene model for the prediction of corresponding metabolite abundance. The star and square symbol beside gene models indicate that the gene model was found to be significantly associated with the metabolite abundance variation in TWAS analysis.

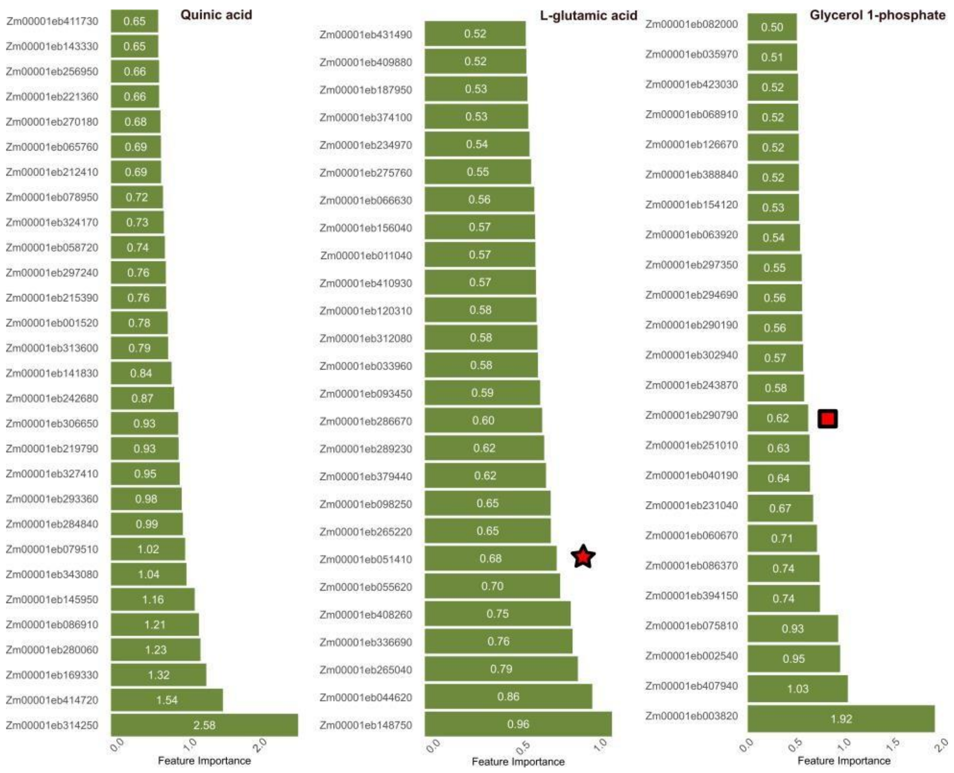

Supplementary Figure S11

Title:
Supplementary Figure S11. Feature importance scores from original and shuffled data from random forest regressor model using gene expression to predict three metabolites abundance in a maize diversity panel.

Legend:
The blue dashed vertical lines indicate the established threshold for significant feature importance. Genes with feature importance higher than the blue threshold in the original data are considered biologically significant to be associated with metabolite abundance.

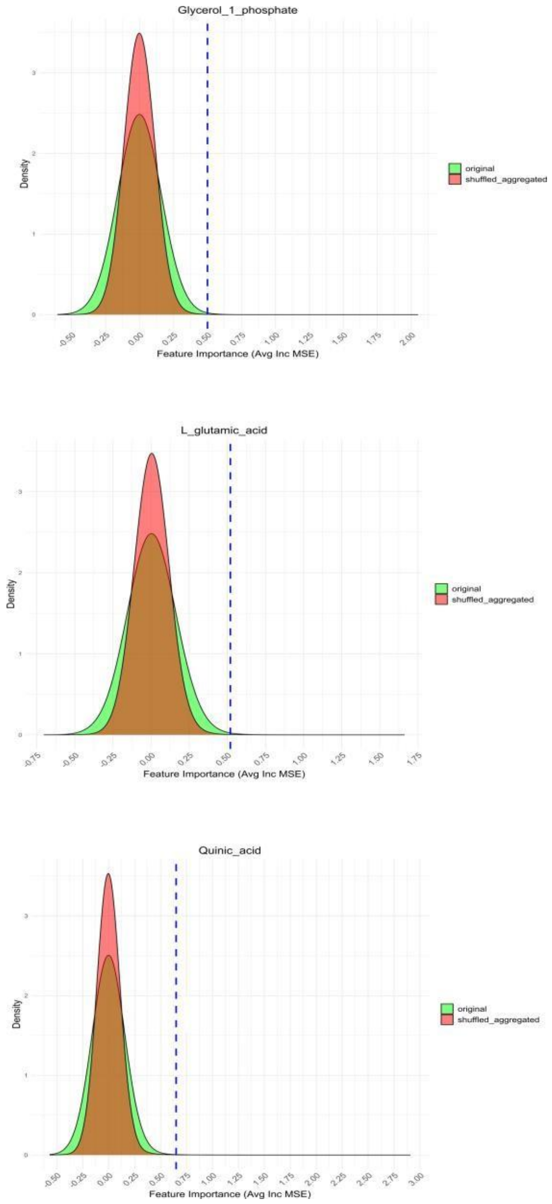

Supplementary Figure S12

Title:
Supplementary Figure S12. Genetic markers associated with 26 metabolites abundances, 28 whole plant phenotypes, 10 latent variables, and 3 photosynthesis variations via resampling model inclusion probability genome-wide association.

Legend:
Each circle's position in the x-axis indicates the position of a given genetic marker on the maize genome, and its position on the y-axis indicates the proportion of resampling runs in which the marker was significantly associated with variation in the trait of interest via FarmCPU GWAS. The plot includes two horizontal dashed lines marking RMIP significance thresholds: the upper red dashed line at 0.20 (indicating SNPs significant in at least 20 out of 100 FarmCPU GWAS) and the lower blue dashed line at 0.10 (indicating SNPs significant in at least 10 out of 100 FarmCPU GWAS). Alternating color horizontal lines along the x-axis indicate the start and end of each maize chromosome.

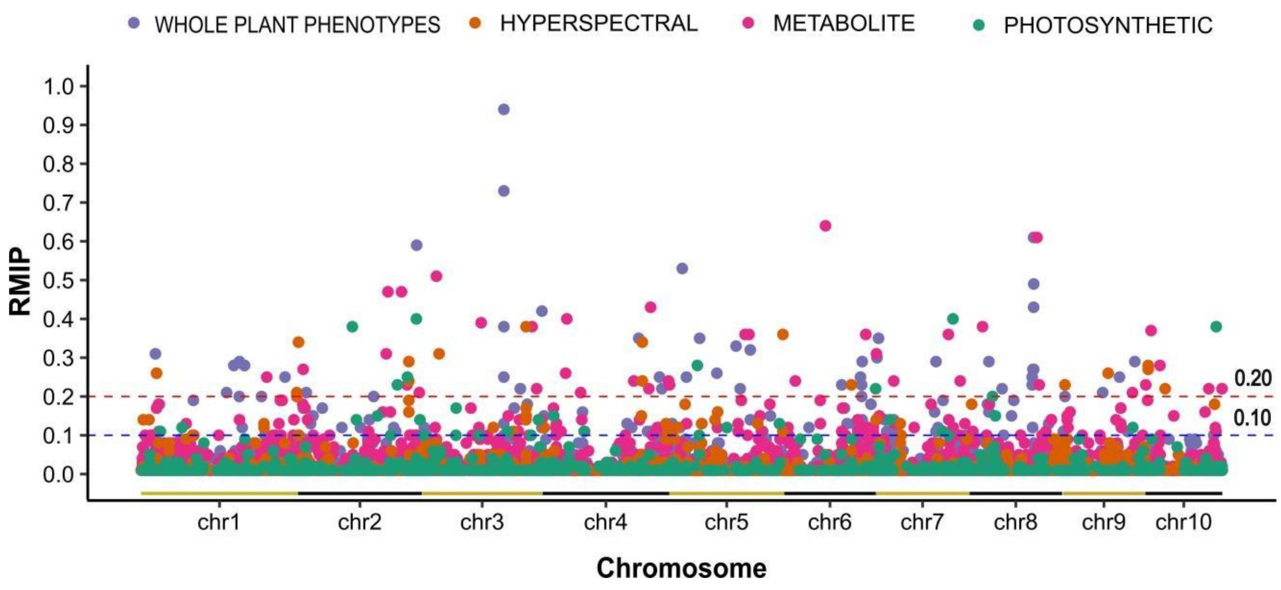

Supplementary Figure S13

Title:
Supplementary Figure S13. Distribution of metabolite abundance across a maize diversity panel.

Legend:
Each histogram represents the frequency distribution of BLUEs (Best Linear Unbiased Estimates) for a specific metabolite. The x-axis denotes metabolite abundance, while the y-axis indicates the count of genotypes. Different metabolites are color-coded for distinction. The dashed red vertical lines represent the upper cutoff values used for outlier removal. The specific cutoffs applied for each metabolite, shown as numerical values next to the red dashed lines.

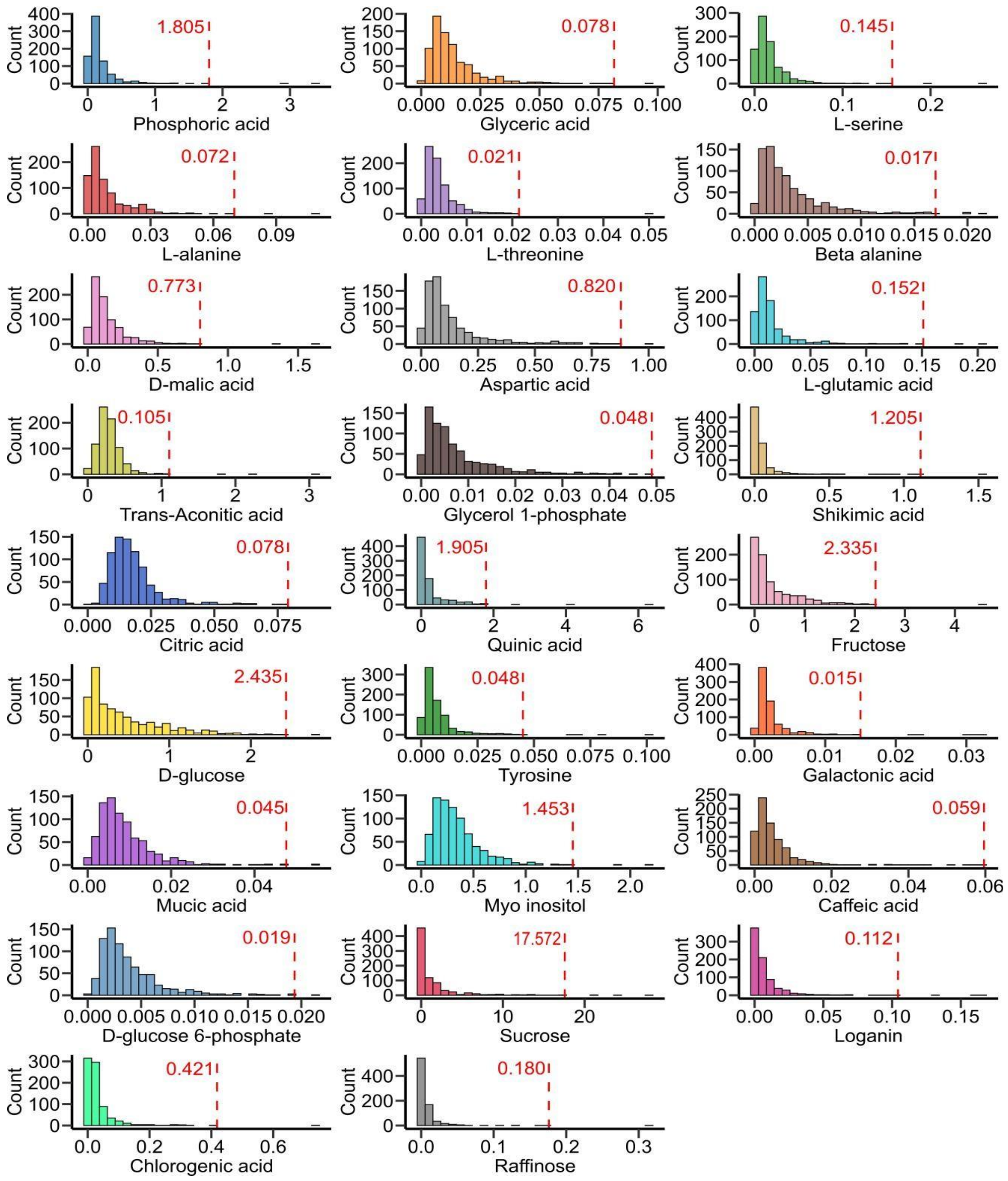

Supplement: Supplementary file 1 — Supplementary Material 1. [file 12864_2025_11580_MOESM1_ESM.zip › Supplementary_Figures.pdf]
